# Short-read fastA files dataset from complexity-reduced genotyping by sequencing data of bacterial isolates from a public hospital in Australia

**DOI:** 10.1016/j.dib.2019.104273

**Published:** 2019-07-16

**Authors:** Berenice Talamantes-Becerra, Jason Carling, Karina Kennedy, Michelle E. Gahan, Arthur Georges

**Affiliations:** a Institute for Applied Ecology, University of Canberra ACT 2601, Australia; b Diversity Arrays Technology Pty Ltd, Canberra ACT, 2617, Australia; c Canberra Health Services, Departments of Microbiology and Infectious Diseases, Canberra Hospital, Yamba Drive, Garran 2605, Australia; d National Centre for Forensic Studies, University of Canberra, ACT, 2617, Australia

**Keywords:** Bacterial pathogens, Genotyping, Short-read sequences

## Abstract

This data article contains short-read sequences (length 30–69 bp) obtained from complexity-reduced genotyping by sequencing (GBS) of 165 samples bacterial isolates from hospital patients in the Australian Capital Territory, between 2013 and 2015. These samples represented 14 bacterial species. Data format is shown as filtered fastA files obtained from an Illumina HiSeq2500 sequencer. The experimental factors of this research used three complexity reduction methods with three combinations of restriction enzymes: *Pst*I with *Mse*I, *Pst*I with *Hpa*II and *Mse*I with *Hpa*II.

**Table:** Specifications table

Subject area	*Microbiology*
More specific subject area	*Medical microbiology*
Type of data	*Short-read sequences*
How data was acquired	*Illumina HiSeq2500 sequencer*
Data format	*Filtered fastA files*
Experimental factors	*Complexity-reduced genotyping by sequencing using three complexity reduction methods on bacterial isolates*
Experimental features	*Combinations of enzymes of PstI with MseI, PstI with HpaII and MseI with HpaII were used.*
Data source location	*Australian Capital Territory, Australia.*
Data accessibility	https://doi.org/10.17632/kp3g7dkzwf.1
Related research article	*Identification of Bacterial Isolates from a Public Hospital in Australia using Complexity-Reduced Genotyping. Talamantes-Becerra, B., Carling, J., Kennedy K*^*.*^*, Gahan, M., Georges, A.* 2019 *Journal of Microbiological methods, Volume 160, May* 2019*, Pages 11–19.*https://doi.org/10.1016/j.mimet.2019.03.016

**Table:** 

**Value of the data** • The data obtained using DArTseq™ complexity-reduced genotyping by sequencing, using three combinations of restriction enzymes, will be useful for comparison of complexity-reduction methods. • The datasets of short-read sequences from bacterial isolates provide an insight to the resolution achieved by complexity-reduced genotyping by sequencing. • The data will be useful for future studies of complexity-reduced genotyping data done on bacterial isolates, as it contains short-reads of the certified reference of Escherichia coli O157 (EDL 933).

## 1 Data

The data are short-read fastA files of complexity-reduced genotyping by sequencing data from bacterial isolates provided by the Microbiology Department of Canberra Public Hospital and short-read fastA files for the certified reference of genomic DNA of *Escherichia coli* [1] O157 (EDL 933) IRMM449 Sigma-Aldrich certified reference standard, GenBank accession number AE005174.2, genome size of 5,639,399 bp [2]. The short-read fastA files were generated by the following method: all samples were processed using the three combinations of restriction enzymes, *Pst*I-*Hpa*II, *Pst*I-*Mse*I and *Mse*I-*Hpa*II. All of the fastA file identification numbers for each complexity-reduction method are shown in Table 1 and Table 2. Data can be accessed using the link above. Each folder contains a Sample_info.csv file which contains the sample names corresponding to each fastA file for each method.

**Table 1:** Filtered fastA file identification numbers for each complexity-reduction method for *E. Coli* O157 EDL 933 certified reference.

Bacterial isolate name	*Filtered fastA file ID*		
	*Mse*I - *Hpa*II	*Pst*I-*Hpa*II	*Pst*I-*Mse*I
*Escherichia coli* O157 EDL933	1577837	1577855	1577845
*Escherichia coli* O157 EDL933	1577838	1577854	1577846
*Escherichia coli* O157 EDL933	1577839	1577853	1577847
*Escherichia coli* O157 EDL933	1577858	1577868	1577859
*Escherichia coli* O157 EDL933	1577862	1577864	1577863
*Escherichia coli* O157 EDL933	1577866	1577860	1577867

**Table 2:** Filtered fastA file identification numbers for each complexity-reduction method for 165 bacterial isolates from a public hospital in Australia.

Species name	Bacterial isolate name	Filtered fastA file ID		
		*Mse*I - *Hpa*II	*Pst*I-*Hpa*II	*Pst*I-*Mse*I
*E. cloacae* complex	002_15CRE063_RA_15P097569	1556133	998829	998931
*K. pneumoniae*	003_16CRE005_Ra_16P005154	1556134	998830	998932
*E. cloacae* complex	004_16CRE025_ME_13P175359	1556135	998831	998933
*K. oxytoca*	005_15CRE004_KD_13P200007	1556136	998832	998934
*E. coli*	006_16CRE008_EK_M442544	1556137	998833	998935
*C. freundii*	007_16CRE004_LP_14P172336	1556138	998834	998936
*K. oxytoca*	008_16CRE002_BB_14P121228	1556139	998835	998937
*C. freundii*	009_16CRE001_CK_14P347252	1556140	998836	998938
*C. freundii*	010_16CRE026_Sa_13P135264	1556141	998837	998939
*E. cloacae* complex	011_16CRE024_WS_12P392018	1556142	998838	998940
*C. amalonaticus*	012_16CRE023_MA_16N015163	1556143	998839	998941
*E. cloacae* complex	013_16CRE020_SR_16P057267	1556144	998840	998942
*E. cloacae* complex	014_16CRE019_SC_12P368071	1556145	998841	998943
*E. asburiae*	015_68ENVIRO_00_14P128774	1556146	998842	998944
*E. asburiae*	016_17ENVIRO_00_14P096931	1556147	998843	998945
*C. freundii*	017_34ENVIRO_00_14P138969	1556148	998844	998946
*C. freundii*	018_38ENVIRO_00_14P174054	1556149	998845	998947
*E. asburiae*	019_21ENVIRO_00_14P121093	1556150	998846	998948
*C. freundii*	020_33ENVIRO_00_14P128774	1556151	998847	998949
*E. asburiae*	021_22ENVIRO_00_14P121095	1556152	998848	998950
*K. oxytoca*	022_23ENVIRO_00_14P121094	1556153	998849	998951
*K. oxytoca*	023_24ENVIRO_00_14P121094	1556154	998850	998952
*C. freundii*	024_37ENVIRO_00_14P167049	1556155	998851	998953
*K. pneumoniae*	025_30ENVIRO_00_14P128773	1556156	998852	998954
*C. freundii*	026_29ENVIRO_00_14P128774	1556157	1001737	1019196
*E. asburiae*	028_27ENVIRO_00_14P121096	1556159	998855	998957
*C. freundii*	029_26ENVIRO_00_14P121096	1556160	998856	1019199
*K. pneumoniae*	030_25ENVIRO_00_14P121096	1556161	998857	1019200
*C. freundii*	031_15CRE003_Sa_13P175773	1556162	1001742	1019201
*K. oxytoca*	033_15CRE055_BR_15P029363	1556164	998860	998962
*E. aerogenes*	034_15CRE087_RA_15N056494	1556165	998861	998963
*K. oxytoca*	035_16CRE029_PD_16P099315	1556166	998862	998964
*K. pneumoniae*	036_16CRE018_DM_12N093483	1556167	998863	998965
*E. cloacae* complex	037_15CRE077_DM_15N043610	1556168	998864	998966
*C. freundii*	038_15CRE032_BD_14P130644	1556169	998865	998967
*K. pneumoniae*	039_15CRE031_WV_14P130878	1556170	998866	998968
*E. cloacae* complex	040_15CRE020_PS_14P102779	1556171	998867	998969
*K. oxytoca*	041_15CRE019_PS_14P102779	1556172	998868	998970
*S. marcescens*	042_15CRE018_PS_14P102779	1556173	998869	998971
*C. freundii*	043_15CRE013_EN_14P015022	1556174	998870	998972
*M. morganii*	044_15CRE052_HM_15P019958	1556175	998871	998973
*K. oxytoca*	045_15CRE060_AL_15P034420	1556176	998872	998974
*E. cloacae* complex	046_15CRE041_CC_14P212313	1556177	998873	998975
*C. freundii*	047_15CRE042_IT_14P215233	1556178	998874	998976
*C. freundii*	048_15CRE044_DA_14P237209	1556179	1001634	1001636
*C. freundii*	049_15CRE047_BS_14P327824	1556180	998884	1019149
*K. oxytoca*	050_16CRE017_CJ_12N011309	1556181	998885	1019155
*E. cloacae* complex	053_16CRE036_MK_16P267082	1556184	1001626	1019173
*M. morganii*	055_16CRE034_SB_16N047000	1556186	998890	1019185
*C. freundii*	056_16CRE032_Sa_16P113450	1556187	998891	1019190
*E. cloacae* complex	057_16CRE016_PP_12P096451	1556188	998892	1019150
*K. pneumoniae*	058_16CRE015_SP_14P413891	1556189	998893	1019156
*K. pneumoniae*	059_16CRE013_SN_16P019291	1556190	998894	1019162
*K. pneumoniae*	060_15CRE049_JE_15P016652	1556191	998895	1019168
*K. pneumoniae*	061_15CRE049_JE__15P016652	1556192	998896	1019174
*P. rettgeri*	062_15CRE69_HM_09P275368	1556193	998897	1019180
*K. pneumoniae*	064_15CRE071_CT_11P278637	1556195	998899	1019191
*K. pneumoniae*	065_15CRE067_SN_15P175158	1556196	998900	1019151
*E. cloacae* complex	066_15CRE008_LM_13P384147	1556197	998901	1019157
*K. oxytoca*	067_15CRE007_TM_13N096416	1556198	998902	1019163
*E. cloacae* complex	069_15CRE012_PV_14P000476	1556200	998904	1019175
*E. cloacae* complex	070_15CRE011_CH_14P323878	1556201	998905	1019181
*E. cloacae* complex	072_15CRE065_DT_14P314366	1556203	998907	1019192
*K. oxytoca*	074_15CRE057_HM_15P019958	1556205	1001769	1001873
*H. alvei*	075_15CRE039_PA_14P177006	1556206	1001770	1001874
*E. cloacae complex*	076_15CRE056_KG_15P008564	1556207	1001771	1001875
*C. freundii*	077_16CRE012_SN_16P019291	1556208	998912	1019176
*K. pneumoniae*	078_16CRE011_PS_14P249549	1556209	998913	1019182
*E. cloacae* complex	079_15CRE074_MJ_15N037855	1556210	998914	1019188
*E. coli*	080_15CRE091_RS_15P341081	1556211	998915	1001879
*E. coli*	081_15CRE091_CM_15P253479	1556212	998916	1019153
*K. pneumoniae*	083_15CRE079_SN_15P174149	1556214	998918	1019165
*E. cloacae* complex	084_15CRE078_DA_15P170231	1556215	998919	1019171
*K. pneumoniae*	085_15CRE054_MR_15P026197	1556216	998920	1019177
*E. coli*	086_15CRE059_AL_15P034420	1556217	998921	1019183
*K. oxytoca*	087_15CRE057_AL_15P033676	1556218	998922	1019189
*E. faecium*	101_2013_ENT01_M457817	1556219	998923	1019194
*E. faecium*	102_2013_ENT04_M463347	1556220	998924	1019154
*E. faecium*	103_2013_ENT06_M470238	1556221	998925	1019160
*E. faecium*	104_2013_ENT08_M479433	1556222	998926	1019166
*E. faecium*	105_2013_ENT12_M488550	1556223	998927	1019172
*E. faecium*	106_2013_ENT16_M496255	1556224	998928	1019178
*E. faecium*	107_2013_ENT18_M499547	1556225	998929	1019184
*E. faecium*	108_2013_ENT21_M507872	1564343	999024	999119
*E. faecium*	110_2013_ENT26_M516964	1564347	999026	999121
*E. faecium*	111_2013_ENT29_M523903	1564349	999027	999122
*E. faecalis*	112_2013_ENT38_M558099	1564351	999028	999123
*E. faecium*	113_2013_ENT43_m565311	1564353	999029	999124
*S. aureus*	114_2013_SAP05_M457547	1564355	999030	999125
*S. aureus*	115_2013_SAP17_M473199	1564357	999031	999126
*S. aureus*	116_2013_SAP21_M477091	1556282	999032	999127
*S. aureus*	117_2013_SAP35_M470525	1564346	999033	999128
*S. aureus*	118_2013_SAP40_M495980	1564348	999034	999129
*S. aureus*	119_2013_SAP41_M495174	1564350	999035	999130
*S. aureus*	121_2013_SAP74_M545002	1556287	999037	999132
*S. aureus*	122_2013_SAP78_M553282	1564356	999038	999133
*S. aureus*	123_2013_SAP81_M555802	1564358	999039	999134
*S. aureus*	124_2013_SAP91_M529003	1564391	999040	999135
*E. faecium*	125_2014_ENT02_M573595	1564361	1019204	1001761
*E. faecium*	126_2014_ENT03_M575704	1564363	1019205	999137
*E. faecium*	127_2014_ENT06_M576744	1564365	999043	999138
*E. faecium*	128_2014_ENT09_M592711_2	1564399	1019207	1001764
*E. faecium*	129_2014_ENT11_M594176	1564401	999045	999140
*E. faecium*	130_2014_ENT13_M600048	1564403	1019209	1001766
*E. faecium*	131_2014_ENT15_M600713	1564405	999047	999142
*E. faecium*	132_2014_ENT17_M601338	1564360	999048	999143
*E. faecium*	133_2014_ENT19_M607507	1564362	999049	999144
*E. faecium*	134_2014_ENT22_M614247	1564364	999050	999145
*E. faecium*	135_2014_ENT24_M615238	1564366	999051	999146
*E. faecium*	136_2014_ENT25_M617159	1564368	999052	999147
*E. faecium*	137_2014_ENT27_M617979	1564370	999053	999148
*E. faecium*	138_2014_ENT30_M621499	1564372	999054	999149
*E. faecium*	139_2014_ENT29_M618981	1564374	999055	999150
*E. faecium*	140_2014_ENT31_M623180	1556258	999056	999151
*E. faecium*	141_2014_ENT32_M626179	1556259	999057	999152
*E. faecium*	142_2014_ENT41_M639056	1556260	999058	999153
*E. faecium*	143_2014_ENT42_M644236	1556261	999059	999154
*E. faecium*	144_2014_ENT45_M648905	1556262	999060	999155
*E. faecium*	145_2014_ENT49_M654113	1556263	999061	999156
*E. faecium*	146_2014_ENT37_M634750	1556264	999062	999157
*E. faecium*	147_2014_ENT39_M635120_2	1556265	999063	999158
*E. faecium*	148_2014_ENT67_M675978	1556266	999064	999159
*E. faecium*	149_2014_ENT68_M678681	1556267	999065	999160
*E. faecium*	150_2014_ENT70_M679959	1556268	999066	999161
*E. faecium*	151_2014_ENT73_M683633	1556269	999067	999162
*E. faecium*	152_2014_ENT74_m686432	1556270	999068	999163
*S. aureus*	153_2014_SAP01_M573439	1556271	999069	999164
*S. aureus*	154_2014_SAP08_M584056	1556272	999070	999165
*S. aureus*	155_2014_SAP15_M593257	1556273	1001635	1001637
*S. aureus*	158_2014_SAP51_M643121	1556276	999074	999169
*S. aureus*	159_2014_SAP54_M655150	1564381	999075	999170
*S. aureus*	160_2014_SAP59_M659228	1556278	1001628	1001632
*S. aureus*	161_2014_SAP60_M659657	1556279	999077	999172
*S. aureus*	162_2014_SAP65_M666867	1556280	999078	999173
*E. faecium*	163_2015_ENT04_M703170	1564389	999079	999174
*E. faecium*	164_2015_ENT06_M707170	1384794	999080	999175
*E. faecium*	165_2015_ENT07_M707211	1384795	999081	999176
*E. faecium*	166_2015_ENT09_M709597	1384796	999082	999177
*E. faecium*	167_2015_ENT11_M712401	1384797	999083	999178
*E. faecium*	168_2015_ENT13_M718043	1384798	999084	999179
*E. faecium*	170_2015_ENT22_M732571	1384800	999086	999181
*E. faecium*	172_2015_ENT24_M738139	1384802	999088	999183
*E. faecium*	173_2015_ENT25_M738435	1384803	999089	999184
*E. faecium*	174_2015_ENT30_M752224	1384804	999090	999185
*E. faecium*	175_2015_ENT31_m752289	1384805	999091	999186
*E. faecium*	176_2015_ENT33_M756888	1384806	999092	999187
*E. faecium*	177_2015_ENT36_M768816	1384807	999093	999188
*E. faecium*	178_2015_ENT37_M771766	1384808	999094	999189
*E. faecium*	179_2015_ENT38_M772755	1384809	999095	999190
*E. faecium*	180_2015_ENT40_M776445	1384810	1001976	999191
*E. faecium*	181_2015_ENT41_M777180	1384811	1001977	1001985
*E. faecium*	182_2015_ENT51_M788361_2	1384812	1001978	1001986
*E. faecium*	183_2015_ENT53_M790234	1384813	1001979	1001987
*E. faecium*	184_2015_ENT56_M803827	1384814	999100	1001988
*S. aureus*	185_2015_SAP01_M692236	1384815	999101	1001989
*S. aureus*	186_2015_SAP11_m703634_2	1384816	999102	999197
*S. aureus*	187_2015_SAP14_m717190	1384817	999103	1001991
*S. aureus*	188_2015_SAP16_m718885	1384818	999104	999199
*S. aureus*	189_2015_SAP31_M742041	1384819	1001629	1001633
*S. aureus*	190_2015_SAP40_M763932	1384820	999106	999201
*S. aureus*	191_2015_SAP47_M769304	1385447	999107	999202
*S. aureus*	192_2015_SAP50_M771826	1564392	999108	999203
*S. aureus*	193_2015_SAP49_M771757	1556299	999109	999204
*S. aureus*	195_2015_SAP75_M804639	1556301	999111	999206
*S. aureus*	196_2015_SAP78_M805765	1384826	999112	999207

## 2 Experimental design, materials and methods

### 2.1 Bacterial strains

A total of 165 samples were isolated from hospital patients and environmental samples in the Australian Capital Territory, between 2013 and 2015. Bacterial samples were provided by the Microbiology Department of Canberra Public Hospital and represented 14 bacterial species. DNA was extracted from all bacterial isolates using a chloroform-isoamyl alcohol method [1].

### 2.2 Library preparation and sequencing

Library preparation followed the DArTseq™ (Canberra, Australia) methods, in which the DNA was digested with pairs of restriction enzymes. The restriction enzymes *Pst*I (5′-CTGCA|G-3′), *Mse*I (5′-TTA|A-3′) and *Hpa*II (5′-CCG|G-3′) were used in combination: *Pst*I with *Mse*I, *Pst*I with *Hpa*II and *Mse*I with *Hpa*II. Bacterial isolates were sequenced to approximately 100,000–150,000 reads per sample. Clustering was done according to Illumina (San Diego CA, US) protocols using a HiSeq SR Cluster Kit V4 recipe v9.0 and HiSeq SR Flow Cell v4. For sequencing, the Flow Cell was loaded according to the Illumina protocols on a HiSeq 2500 sequencer, using HiSeq SBS kit v4 for a total of 77 cycles [3].

Sequences can be downloaded as filtered fastA files. Table 1 shows the filtered fastA file identification numbers for six technical replicates of each complexity-reduction method for the certified reference material of *E. Coli* O157 EDL 933. Table 2 indicates the identification name for each bacterial isolate along with the filtered fastA file identification numbers for all 165 bacterial isolates for the three combinations of restriction enzymes used.

### 2.3 Production of data files

Raw data obtained from the sequencer in the form of fastQ files were demultiplexed using the DArTseq™ primary data processing pipeline. It produced one fastQ file for each sample assayed. Filtering of reads was done in two steps on Phred score [4] as described in Georges et al. [3]. The barcode was removed from the reads, leaving fragments of 69 bp. The fastQ files were condensed into a fastQcol files which contained each unique sequence present in the original fastQ file, along with the respective read counts and the mean quality score at each base [5]. Unique sequences contained SeqIndex identifiers. Adapters were trimmed leaving fragments up to 69 bp. Fragments with less than 30 bp were removed. The datafiles consist of fastA files produced by the method described above.

## Funding

This research did not receive any specific grant from funding agencies in the public, commercial, or not-for-profit sectors.
